# T-bet expression in intratumoral lymphoid structures after neoadjuvant trastuzumab plus docetaxel for HER2-overexpressing breast carcinoma predicts survival

**DOI:** 10.1038/bjc.2011.261

**Published:** 2011-07-12

**Authors:** S Ladoire, L Arnould, G Mignot, L Apetoh, C Rébé, F Martin, P Fumoleau, B Coudert, F Ghiringhelli

**Affiliations:** 1 Department of Medical Oncology, Georges François Leclerc Center, Centre de Recherche INSERM 866, Faculté de Médecine, 7 Boulevard Jeanne d’Arc, Dijon 21000, France; 2 Institut National de la Santé et de la Recherche Médicale, Avenir Team INSERM, CRI-866 University of Burgundy, Dijon, France; 3 Department of Pathology and Biology of Tumors, Georges François Leclerc Center, Dijon 21000, France

**Keywords:** T-bet, breast cancer, HER2, trastuzumab, docetaxel

## Abstract

**Background:**

In HER2-overexpressing breast cancer, accumulating preclinical evidences suggest that some chemotherapies, like trastuzumab, but also taxanes, are able to trigger a T helper 1 (Th1) anticancer immune response that contribute to treatment success. T helper 1 immune response is characterised by the expression of the transcription factor T-bet in CD4 T lymphocytes. We hypothesised that the presence of such T cells in the tumour immune infiltrates following neoadjuvant chemotherapy would predict patient survival.

**Methods:**

In a series of 102 consecutive HER2-overexpressing breast cancer patients treated by neoadjuvant chemotherapy incorporating antracyclines or taxane and trastuzumab, we studied by immunohistochemistry the peritumoral lymphoid infiltration by T-bet+ lymphocytes before and after chemotherapy in both treatment groups. Kaplan–Meier analysis and Cox modelling were used to assess relapse-free survival (RFS).

**Results:**

Fifty-eight patients have been treated with trastuzumab–taxane and 44 patients with anthracyclines-based neoadjuvant chemotherapy. The presence of T-bet+ lymphocytes in peritumoral lymphoid structures after chemotherapy was significantly more frequent in patients treated with trastuzumab–taxane (*P*=0.0008). After a median follow-up of 40 months, the presence of T-bet+ lymphocytes after neoadjuvant chemotherapy confers significantly better RFS (log-rank test *P*=0.011) only in patients treated with trastuzumab–taxane. In this population, multivariate Cox regression model showed that only the presence of T-bet+ lymphocytes in peritumoral lymphoid structures after neoadjuvant chemotherapy was independently associated with improved RFS (*P*=0.04).

**Conclusion:**

These findings indicate that the tumour infiltration by T-bet+ Th1 lymphocytes following neoadjuvant trastuzumab–taxane may represent a new independent prognostic factor of improved outcome in HER2-overexpressing breast carcinoma.

Neoadjuvant chemotherapy has allowed oncologists to assess treatment response, and to tailor their treatments. Pathologic complete response (pCR) after neoadjuvant chemotherapy has been described as a strong indicator of chemosensitivity, justifying its use as a surrogate marker of survival (Kuerer *et al*, 1999). Although chemotherapy mainly acts directly by cytotoxic effect on tumour cells, recent works indicate that some chemotherapies also have the ability to harness the host's immune system to fight against its tumour (Zitvogel *et al*, 2008). For example, in HER2-overexpressing breast cancer, trastuzumab, an anti-HER2 monoclonal antibody, has drastically improved patient prognosis in adjuvant and neoadjuvant (Buzdar *et al*, 2005; Piccart-Gebhart *et al*, 2005; Romond *et al*, 2005; Untch *et al*, 2010) setting when combined with chemotherapy. Trastuzumab is often associated with taxanes in synergistic chemotherapy regimens. Trastuzumab kills cancer cells by blocking intracellular transduction signals, but also restrain their growth by activating the host's immune system through antibody-dependent cellular cytotoxicity (ADCC) (Arnould *et al*, 2006). This event leads to T helper 1 (Th1) activation of T cells, an adaptative immune response characterised by T cells production of interferon-*γ* (IFN-*γ*), and implicated in cancer growth control (Dhodapkar *et al*, 2002). Moreover, recent works indicated that taxanes could also exert immunostimulatory effects against breast cancer (Lee *et al*, 2000; Tsavaris *et al*, 2002; Carson *et al*, 2004), notably by inducing Th1 immune response. There is increasing evidence that the development of Th1 adaptive immunity is associated with improved outcome in various cancer types (Zhang *et al*, 2003; Galon *et al*, 2006; Roepman *et al*, 2009). T-bet (TBX21) is a Tbox transcription factor known to be crucial for the development of effector Th1 CD4 T cells (Mullen *et al*, 2001), and the most specific marker of this T-cell subset.

The current study was undertaken to analyse the prognostic role of Th1 polarised T cells expressing T-bet in intratumoral lymphoid structures, in patients undergoing neoadjuvant chemotherapy incorporating or not trastuzumab and taxane for HER2-overexpressing breast cancer. Interestingly, this study reveals that the presence of T-bet+ lymphocytes in intratumoral lymphoid structures after neoadjuvant chemotherapy is significantly more frequent after trastuzumab–taxane-based chemotherapy, and could be a new prognostic biomarker of improved survival in this group of patients.

## Patients and methods

### Patients

We retrospectively studied cancer-tissue specimens from 660 patients who underwent neoadjuvant chemotherapy for non-metastatic breast cancer at the Georges François Leclerc Cancer Center, Dijon, and Sainte Marie Private Hospital, Chalon sur Saone, France, from January 1981 to November 2008. In all these patients, HER2 status was determined using the Herceptest scoring method, considering only three-grade score as determining HER2-positive tumours. For patients with two-grade score, a chromogenic *in situ* hybridisation was performed to determine HER2 overexpression. According to this test, we only recruited in this study the 102 consecutive HER2-overexpressing breast cancers in which we have paraffin-embedded initial tumour biopsies and surgical specimens for immunohistochemical study. The study was approved by the ethical local committee, and patients gave written informed consent for the use of samples from their tumours for future investigations at the time of the diagnosis. Histoprognostic grade was defined according to the modified Bloom and Richardson method by Elston and Ellis. The steroid hormone receptors statuses were determined using immunochemistry. Chemotherapy treatment consisted of an anthracycline-based regimen from 1981 to 2000: FEC 100 (epirubicin 100 mg m^–2^, cyclophosphamide 500 mg m^–2^, and 5-fluorouracil 500 mg m^–2^) or FAC (adriamycine 50 mg m^–2^, cyclophosphamide 500 mg m^–2^, and 5-fluorouracil 500 mg m^–2^). From 2001 to 2007, the regimen consisted in trastuzumab (2 mg kg^–1^ per week) associated with chemotherapy by docetaxel 100 mg m^–2^ or by docetaxel 75 mg m^–2^ + carboplatin AUC 6. After six cycles of chemotherapy, breast surgery (conservative whenever possible) and axillary lymph node dissection were mandatory 3–5 weeks after the last cycle. Adjuvant radiotherapy was administrated in all patients and postsurgical hormonal therapy was administrated in patients with hormone receptor-positive tumours. Histological response was determined on surgical specimens: breast tissue without residual malignant epithelial invasive tumour, and associated with no microscopic evidence of tumour cell in axillary specimens, was considered as pCR.

### Immunohistochemical labelling

Immunohistochemistry used monoclonal antibodies against the T-bet (TBX21) transcription factor (Santa Cruz, Heidelberg, Germany, clone 4B10). Antigen retrieval was carried out by heating slides for 15 min at 95°C in 1 mmol l^–1^ EDTA. Labelling was detected using the Dako Envision system (Dako, Trappes, France). The stained arrays were counterstained with haematoxylin and mounted in Aquamount (Dako). Positive and negative staining controls were carried out with paraffin tonsil sections using T-bet monoclonal antibody and an isotype-matched negative control antibody.

### Presence of T-bet+ lymphocytes

The presence or absence of T-bet+ lymphocytic infiltration was evaluated by two independent physicians (FG and SL) in the lymphoid peritumoral areas of the entire tissue section. All samples were previously anonymised and blinded to the clinicopathologic data. For each tissue section, the presence or absence of T-bet+ cells were established after the analysis of 20 high power fields (20 × ). T-bet induction was defined by the absence of T-bet+ T cells on the initial tumour specimen, associated with apparition of T-bet+ lymphocytes in intratumoral lymphoid structures on surgical specimen after completion of chemotherapy in the same patient. The results of the analyses conducted by each independent pathologist were subsequently compared (*κ* score was 0.96). Discrepancies between the two observers were reviewed jointly to reach a consensus. As a number of tumour without T-bet+ cells could be found, we dichotomise patient between the presence and absence of T-bet+ cells. When T-bet+ cells were found present in the tumour, we always observed these cells in tertiary lymphoid organs closed to tumour bed. These cells could be enumerated and represent around 3–15 cells per lymphoid structure.

### Statistical analyses

Qualitative variables were described as frequencies and percentages. The association of variables was evaluated with the *χ*^2^ test. The Wilcoxon test was used to compare non-continuous variables in paired samples as appropriate. Relapse-free survival (RFS) was calculated from the date of diagnosis until the date of relapse (local or metastatic). Alive or dead patients without relapse were censored at the last follow-up. Follow-up was calculated using reverse Kaplan–Meier method. Relapse-free survival probabilities were estimated using Kaplan–Meier method and were compared by the log-rank test. The hazards ratios (HRs) with 95% confidence interval (CI) were calculated using univariate Cox proportional hazards regression modelling. All variables with a univariate Cox *P*-value ⩽0.20 were eligible for multivariate analyses. Correlations between co-variables were first tested for eligible variables. To prevent collinearity, when two variables were significantly correlated, one variable was retained according to its clinical relevance or to the value of the likelihood ratio. Finally, multivariate Cox proportional hazards regression modelling was applied to assess independent prognosis effect for RFS. All reported *P*-values are two sided. The statistical significance level was set at *P*<0.05. Analyses were performed using STATA (version 11.0, Stata Corp, College Station, TX, USA).

## Results

### Patient characteristics

In total, 102 patients with a confirmed HER2-overexpressing breast cancer were included in this analysis. Patient and tumour characteristics are reported in Table 1. Half of all patients presented with stage T3 or T4 tumour and 72% had clinically detectable axillary lymph node involvement at diagnosis. Half the patients presented with high tumour grade (SBR III) and/or negative oestrogen receptor. Neoadjuvant chemotherapy was given every 3 weeks for a total of six cycles, and consisted of an anthracycline-based regimen in 44 cases, and trastuzumab–taxane-based regimen in 58 cases. Classic histological analysis of surgical specimens revealed a pCR in 32 cases (31%). Median follow-up period was 40 months (range 7–144). Kaplan–Meier curves of pCR *vs* no-pCR groups for both cohorts were presented in Supplementary Figure 1.

Comparisons between the two groups of treatment reveal that patients treated after 2001 with trastuzumab–taxane-based neoadjuvant chemotherapy had significantly less high-grade tumours and axillary lymph node involvement. Pathologic complete response was more frequently achieved in trastuzumab–taxane group (41% *vs* 18% *P*=0.02) (Table 1).

**Figure 1:**
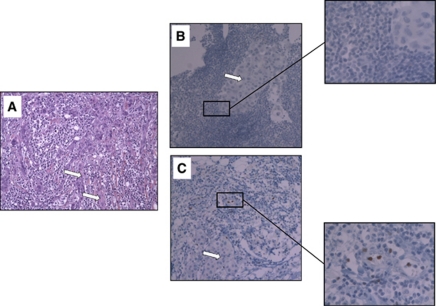
(**A**) Haematoxylin–eosin–safran labelling of an HER2-overexpressing breast cancer. White arrows indicate tumour islets and the black arrow the paratumoral lymphoid structure (Magnification × 10). Immunohistochemical T-bet staining of paraffin-embedded breast cancer overexpressing HER2. (**B**) Representative tumour with lymphoid structure devoid of T-bet+ lymphocyte infiltration. (**C**) Representative tumour with lymphoid structure with T-bet+ lymphocyte infiltration. White arrows show tumour islets (magnification × 10). A detail of lymphoid infiltrate is shown on × 40 magnification.

The presence of T-bet+ lymphocytes were analysed in the whole tumour and in the peritumoral areas. T-bet + cells were only located in peritumoral lymphoid structures (Figure 1). Before chemotherapy, T-bet+ cells were rare and the percentage of positive tumour did not differ between the two treatment groups (*P*=0.99). After chemotherapy, our analysis revealed an absence of T-bet+ cells in 66 tumours, the presence of 3–5 T-bet+ cells in 25 tumours and presence of more than five T-bet+ cells in 11 tumours. Importantly, we did not observe any correlation between residual tumour size and level of T-bet infiltration (low *vs* high infiltration). We have, therefore, decided to use only a dichotomic classification (absence *vs* presence of T-bet+ cells). Interestingly, after neoadjuvant chemotherapy, 50% of patients treated with trastuzumab–taxane had T-bet+ cells infiltration in peritumoral lymphoid structures, *vs* only 16% of patients treated with antracyclines (*P*=0.0008). T-bet induction was significantly more frequent in patients treated with trastuzumab and taxane (*P*=0.01) (Table 1).

### Association of outcomes with immunological findings

**Figure 2:**
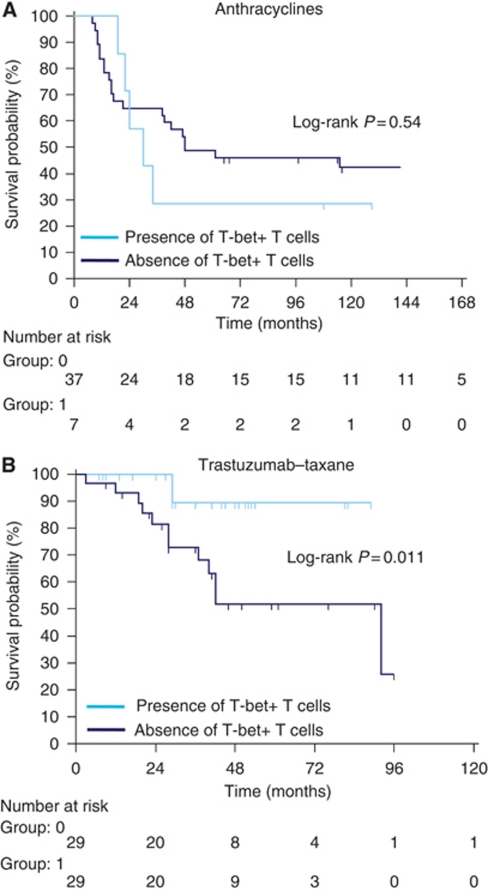
Presence of T-bet+ T cells in peritumoral lymphoid structure predicts better RFS in patients treated with neoadjuvant taxanes–trastuzumab-based neoadjuvant chemotherapy. Kaplan–Meier curves for RFS stratified according to the presence or absence of T-bet+ T cells after neoadjuvant chemotherapy in (**A**) patients treated with anthracyclines, (**B**) patients treated with taxanes–trastuzumab. *P*-values were calculated using the log-rank test.

In patients treated with anthracyclines, univariate Cox proportional analysis indicated that neither the presence of T-bet+ cells before, nor after neoadjuvant chemotherapy, nor T-bet+ cell induction were associated with an increased risk of relapse (RFS). By contrast, in patients treated with trastuzumab–taxane, the presence of T-bet+ cells in peritumoral lymphoid structures after chemotherapy (*P*=0.02; HR: 5.6; 95% CI: 1.25–25) and T-bet+ cells induction (*P*=0.04; HR: 6.7; 95% CI: 1.08–38) were associated with a better RFS (Table 2). The presence of T-bet+ cells after neoadjuvant chemotherapy confers significantly better RFS (log-rank test *P*=0.011) only in patients treated with trastuzumab and taxane (Figure 2A and B). We did not observe any significant correlation between T-bet induction and pCR thus indicating that T-bet induction is a prognostic factor independent of the pathological response. In this population, multivariate Cox regression model showed that only the presence of T-bet+ cells in peritumoral lymphoid structures after neoadjuvant chemotherapy was independently associated with improved RFS (*P*=0.04; HR: 4.76; 95% CI: 1.07–20) (Table 3). Inclusion of pCR in the multivariate model did not change the capacity of T-bet expression to predict RFS. Correlation between T-bet presence after chemotherapy and classical clinical prognostic factors demonstrates that no classical prognostic factors are linked to T-bet expression after chemotherapy (Table 4).

## Discussion

This study highlights the importance of host's immune response in the prognosis of breast cancer, and the interaction between chemotherapy regimen and the immune system in this setting. In our series, neoadjuvant chemotherapy incorporating taxanes and trastuzumab seems to influence the apparition of a Th1 immune response in the intratumoral lymphoid structures in contrast to anthracyclines. Moreover, the presence of these T-bet+ lymphocytes after neoadjuvant trastuzumab–taxane chemotherapy is associated with better RFS in this population.

In recent years, convincing data supporting the prognostic role of immune response against cancer was emerging (Zitvogel *et al*, 2006). In cancer setting, host protection is largely afforded via the generation of Th1 response, in which the transcription factor T-bet is playing a key role (Mullen *et al*, 2001). In patients with colorectal carcinoma, the presence of mRNA encoding molecules expressed by Th1 cells, such as T-bet, correlates with reduced metastatic invasion and increased survival (Galon *et al*, 2006). However, the prognostic role of T-bet+ lymphocytes in breast cancer has not been yet investigated.

Trastuzumab plus docetaxel is an approved anticancer regimen that is used worldwide for the treatment of HER2-overexpressing breast cancer notably in neoadjuvant setting (Coudert *et al*, 2007). Trastuzumab contains an IgG1 Fc structure. When the Fc*γ* receptor on immune effector cells detects the Fc portion of the antibody bound to target cells, immune effector cells are recruited to attack the target cells (Lazar *et al*, 2006). *In vitro*, this process is termed as ADCC. Laboratory studies have shown that HER2-overexpressing breast cancer cell lines are susceptible to ADCC in the presence of trastuzumab (Carson *et al*, 2001; Parihar *et al*, 2002; Gennari *et al*, 2004), and *in vivo* activity of trastuzumab has been correlated with significantly increased number of peritumoral lymphocytes and monocytes and *in vitro* ADCC (Gennari *et al*, 2004; Arnould *et al*, 2006). Additional studies have indicated that NK cells are keys for trastuzumab-mediated ADCC (Cooley *et al*, 1999; Clynes *et al*, 2000; Mimura *et al*, 2005). Nonetheless, activated NK cells, by their secretion of INF-*γ* could activate macrophages, which in turn produce interleukin-12 (IL-12), which is playing a key role in Th1 polarisation (Manetti *et al*, 1993). Thus, induction of T-bet, the pathognomic marker of Th1 polarisation could be a surrogate marker of the immunological effects of trastuzumab, but also of taxanes. Indeed, recent works indicated that taxanes could exert immunostimulatory effects against breast cancer. Thus, paclitaxel is able to stimulate the secretion by macrophages of proinflammatory and Th1 cytokines such as IL-1*β* or IL-12 (Mullins *et al*, 1998; Chan and Yang, 2000). Preclinical studies have shown that breast cancer bearing mice responding to docetaxel chemotherapy harboured strong tumoral infiltration with T lymphocytes and NK cells (Mason *et al*, 2001), and these observations have been also reported in breast cancer patients (Tong *et al*, 2000; Demaria *et al*, 2001). In 227 breast cancer patients, Carson *et al* (2004) demonstrated that T-cell activation was significantly higher in patients receiving taxanes compared with non-containing taxanes regimens. In patients with metastatic breast cancer, Tsavaris *et al* (2002) found that patients responding to paclitaxel or docetaxel-based chemotherapy harboured a significant increase in Th1 cytokine serum levels (IL-2 and INF-*γ*). All these immunological findings may partially explain the synergistic activity of trastuzumab and docetaxel in the treatment of HER2-overexpressing breast cancer and the excellent clinical outcome afforded by this combination in neoadjuvant setting (Guiu *et al*, 2011). Thus, efforts addressing the mechanisms mediating trastuzumab and taxanes immunological activity, and biomarkers of this activity would allow for optimal use of this treatment for patients with HER2-overexpressing breast cancer.

In order to prove that T-bet induction was none specific than trastuzumab regimen, we used a control group of patients treated with anthracyclines-based chemotherapy. Preclinical data obtained in mouse have suggested the involvement of immune system in the antitumour response mediated by anthracyclines (Apetoh *et al*, 2007). However, in the present study, we did not observe a significant T-bet induction in intratumoral lymphoid structures after chemotherapy in patients treated with an anthracycline-based regimen. The explanation for this discrepancy might lie in the fact that anthracylines primarily activate CD8 (and not CD4) T cells to produce IFN-*γ* in mouse models and the transcription factor T-bet drives IFN-*γ* secretion in CD4 T cells but not in CD8 T cells. These results thus suggest that the use of T-bet expression as a biomarker of the potential immunologic effects of anthracyclines might not be appropriate. In addition, since taxanes are always associated with trastuzumab, we could not determine if the immune response induced by taxanes plus trastuzumab is due to taxanes, trastuzumab, or their combination. However, the previous results obtained by Zitvogel *et al*, suggesting the inability of docetaxel to induce an immunogenic cell death, would support the hypothesis that the combination of taxanes plus trastuzumab is required to elicit an immune response against cancer.

One possible bias of our study is the selection of the control group. Because of the retrospective design of the study, the control group might have some differences in prognostic variables compared with the taxanes plus trastuzumab group. These differences might have affected our observed results.

In conclusion, this study highlights another possible proof-of-principle of immune modulation by the trastuzumab–taxane combination in HER2-overexpressing breast carcinoma, and suggests that T-bet induction or the presence of T-bet+ lymphocytes in intratumoral lymphoid structures after neoadjuvant chemotherapy could be a new biomarker of the long-term efficacy of this regimen, which could help to select high-risk patients for additional therapies after neoadjuvant chemotherapy. In a previous work, we have demonstrated that the CD8/Foxp3 ratio in tumour bed after chemotherapy is associated with a favourable outcome. This ratio is applicable to both patients treated with anthracycline and patients treated with trastuzumab plus taxanes. T-bet expression is a predictor of outcome only in patients treated with trastuzumab plus taxanes. Further studies are warranted to test if the association of CD8, Foxp3 and T-bet labelling will further optimise the prognostic prediction in patients treated with trastuzumab plus taxanes. The issue of whether T-bet might become a clinically usable prognostic marker in breast carcinoma will need to be evaluated in other larger series of patients, and analysis will have to be stratified on breast molecular subtype, and on chemotherapy regimen.

## Figures and Tables

**Table 1:** Patient and tumour characteristics (*n*=102)

	**Patients treated with anthracyclines (*n*=44)**		**Patients treated with taxane–trastuzumab (*n*=58)**		
	***N***	**%**	***N***	**%**	***P***
*Age (years)*					
<50	24	54	32	55	
⩾50	20	46	26	45	0.89
					
*Tumour initial stage*					
T2	18	41	36	62	
T3–T4	26	59	22	38	0.06
					
*Axillary nodal status*					
Positive	38	86	35	60	
Negative	6	14	23	40	0.01
					
*Tumour grade*					
I+II	18	41	37	64	
III	26	59	21	36	0.04
					
*Oestrogen receptor*					
Positive	17	39	33	57	
Negative	27	61	25	43	0.10
					
*Pathological complete response*					
Yes	8	18	24	41	
No	36	82	34	59	0.02
					
*T-bet+ infiltrates before CT*					
Yes	9	21	13	22	
No	35	79	45	78	0.99
					
*T-bet+ infiltrates after CT*					
Yes	7	16	29	50	
No	37	84	29	50	0.0008
					
*T-bet+ induction*					
Yes	6	14	22	38	
No	38	86	36	62	0.01
					
*Relapse*					
Yes	26	59	14	24	
No	18	41	44	76	0.0007
					
*Death*					
Yes	23	52	2	4	
No	21	48	56	96	<0.0001

Abbreviation: CT=chemotherapy.

**Table 2:** Univariate analysis for factors associated with relapse-free survival in patients treated with neoadjuvant chemotherapy

	**Anthracyclines (*n*=44)**			**Taxane–trastuzumab (*n*=58)**		
	**Univariate HR**	**95% CI**	***P***	**Univariate HR**	**95% CI**	***P***
*Age (years)*						
⩾50	1			1		
<50	0.61	0.28–1.31	0.20	2.9	0.82–10.8	0.09
						
*Tumour initial stage*						
T2	1			1		
T3–T4	1.10	0.51–2.41	0.79	1.91	0.64–5.7	0.24
						
*Axillary nodal status*						
Negative	1			1		
Positive	1.32	0.40–4.40	0.64	1.62	0.50–5.25	0.42
						
*Tumour grade*						
I+II	1			1		
III	0.67	0.31–1.45	0.30	1.49	0.50–4.40	0.47
						
*Oestrogen receptor*						
Positive	1			1		
Negative	1.19	0.55–2.60	0.65	2.8	0.92–8.3	0.07
						
*Pathological complete response*						
Yes	1			1		
No	2.32	0.69–7.60	0.17	2	0.62–6.30	0.25
						
*T-bet+ infiltrates before CT*						
Yes	1			1		
No	2.6	0.80–9.1	0.11	1.51	0.34–6.7	0.58
						
*T-bet+ infiltrates after CT*						
Yes	1			1		
No	0.74	0.28–1.96	0.55	5.6	1.25–25	0.02
						
*T-bet+ induction*						
Yes	1			1		
No	0.55	0.20–1.49	0.24	6.7	1.08–38	0.04

Abbreviations: CI=confidence interval; CT=chemotherapy; HR=hazard ratio.

**Table 3:** Multivariate analysis for factors associated with relapse-free survival in patients treated with taxane–trastuzumab neoadjuvant chemotherapy (*n*=58)

	**Multivariate HR**	**95% CI**	***P***
*Age (years)*			
⩾50	1		
<50	1.75	0.37–8.22	0.47
			
*Oestrogen receptor*			
Positive	1		
Negative	1.88	0.51–7.14	0.34
			
*T-bet+ infiltrates after CT*			
Yes	1		
No	4.76	1.07–20	0.04

Abbreviations: CI=confidence interval; CT=chemotherapy; HR=hazard ratio.

**Table 4:** Frequency of prognostic clinicopathological characteristics of the 58 patients treated with trastuzumab–taxane CT, according to the presence or the absence of T-bet+ cells after CT

	**Presence of T-bet+ cells**		**Absence of T-bet+ cells**		
	***N***	**%**	***N***	**%**	***P***
*Age (years)*					
<50	14	49	18	62	
⩾50	15	51	11	38	0.43
					
*Tumour initial stage*					
T2	21	72	15	51	
T3–T4	8	28	14	49	0.17
					
*Axillary nodal status*					
Positive	18	62	17	58	
Negative	11	38	12	42	0.99
					
*Tumour grade*					
I+II	19	65	18	62	
III	10	35	11	38	0.99
					
*Oestrogen receptor*					
Positive	18	62	15	51	
Negative	11	38	14	49	0.59
					
*Pathological complete response*					
Yes	15	51	9	31	
No	14	49	20	69	0.18

Abbreviation: CT=chemotherapy.
