# Supplementary material for: T-bet expression in intratumoral lymphoid structures after neoadjuvant trastuzumab plus docetaxel for HER2-overexpressing breast carcinoma predicts survival
Source: Br J Cancer. 2011 Jul 12;105(3):366–71. doi: 10.1038/bjc.2011.261 (PMC3172914; doi:10.1038/bjc.2011.261)

## Slide 1
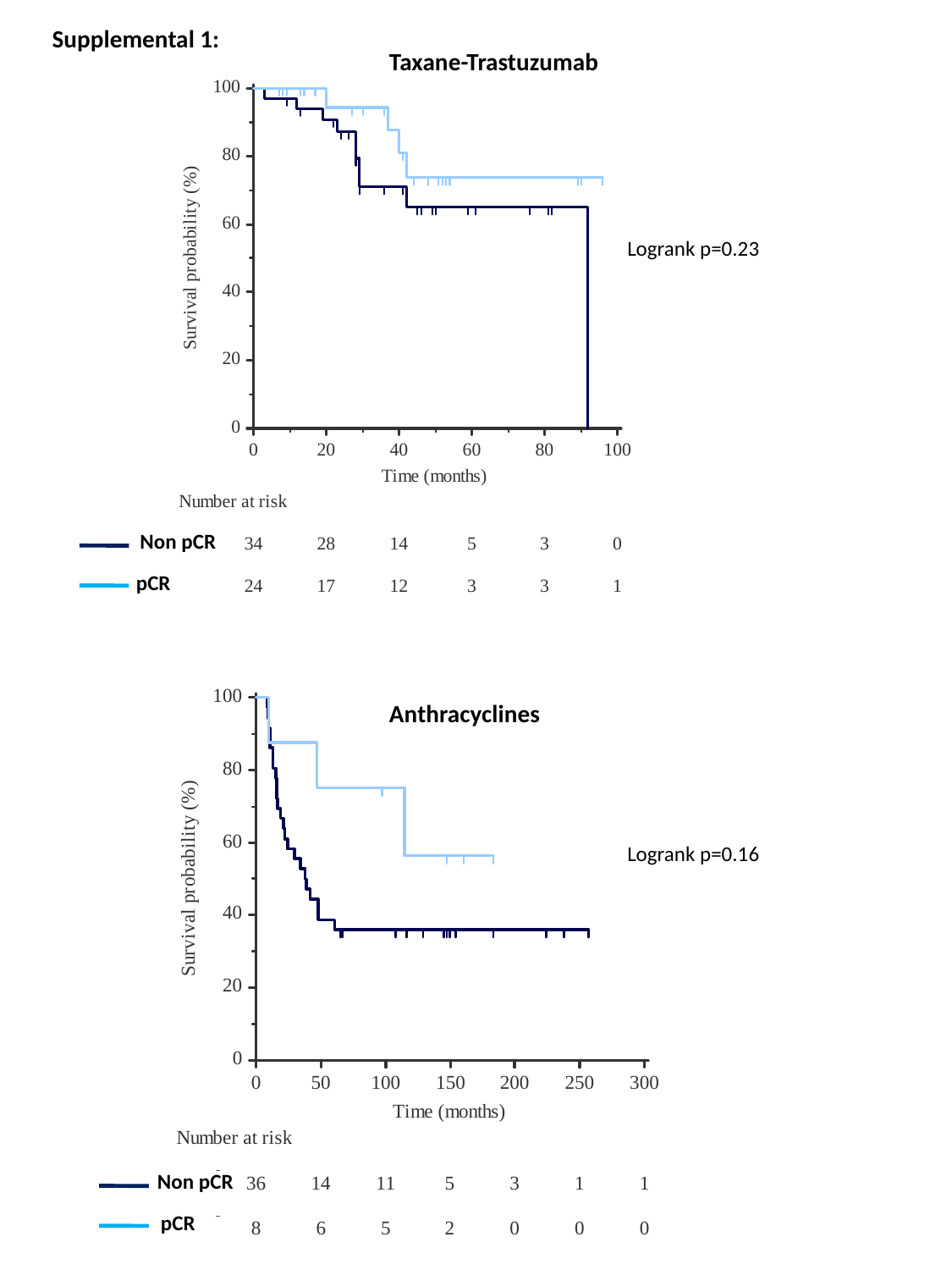

Supplemental 1:
Taxane-Trastuzumab
Logrank p=0.23
Non pCR
pCR
Anthracyclines
Logrank p=0.16
Non pCR
 pCR

## Slide 2
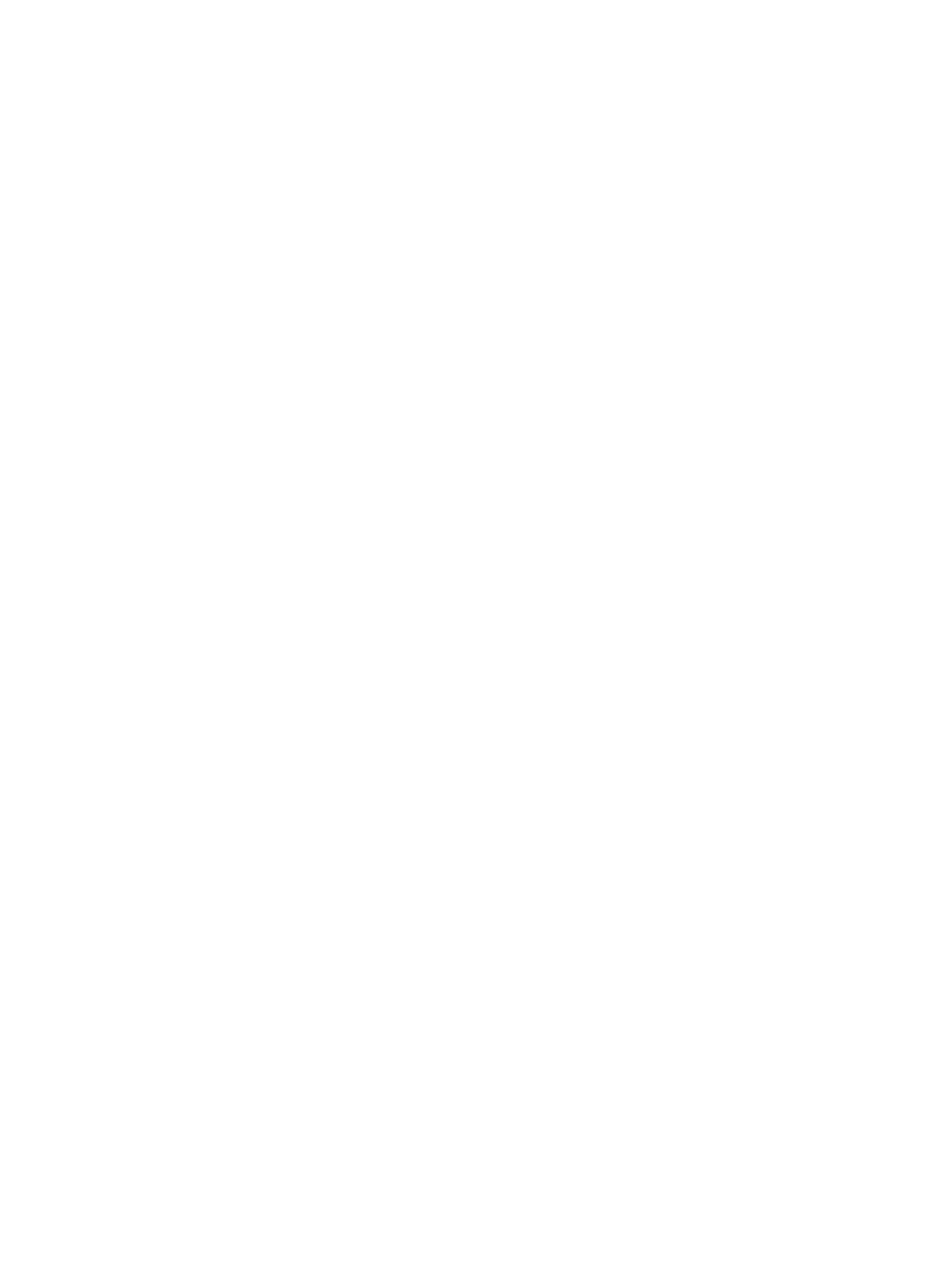

Supplement: Supplementary Figure 1 [file bjc2011261x1.ppt]
