# Supplementary material for: T-bet expression in intratumoral lymphoid structures after neoadjuvant trastuzumab plus docetaxel for HER2-overexpressing breast carcinoma predicts survival
Source: Br J Cancer. 2011 Jul 12;105(3):366–71. doi: 10.1038/bjc.2011.261 (PMC3172914; doi:10.1038/bjc.2011.261)
Supplement: Supplementary Figure legend [file bjc2011261x2.doc]

**Supplementary Figure 1 :** Kaplan-Meier curves for RFS stratified according to presence or absence of pCR after neoadjuvant chemotherapy in patients treated with taxanes-trastuzumab (upper panel) and patients treated with anthracyclines (lower panel). *P* values were calculated using the log-rank test.
